# Supplementary material for: Patterns of Intron Gain and Loss in Fungi
Source: PLoS Biol. 2004 Nov 30;2(12):e422. doi: 10.1371/journal.pbio.0020422 (PMC532390; doi:10.1371/journal.pbio.0020422)
Supplement: Table S1 — Also available at http://genes.mit.edu/NielsenEtAl/. (4.3 MB ZIP). [file pbio.0020422.st001.zip › NielsenEtAl/html/798.html]

AN2998.1.NCU03877.1.MG01014.1.FG10267.1


```
 CLUSTAL W (1.82) Multiple Sequence Alignments - Introns Inserted


Sequence 1: NCU03877.1	941 aa
Sequence 2: MG01014.1	1062 aa
Sequence 3: AN2998.1	665 aa
Sequence 4: FG10267.1	1020 aa
Alignment Length: 1152 aa
Number Identitical Residues: 468 aa
Alignment Score (without introns) 23895


MG01014.1 	MRRCSRTTSLHPNSRSTSLARFSSLAAARHTLNLQSSSNDSSAIRLQTEPAAATAATHPG
NCU03877.1	------------------------------------------------------------
FG10267.1 	------------------------------------------------------------
AN2998.1  	------------------------------------------------------------
          	                                                            

MG01014.1 	RYRLPSLTCSPDPRATLRGPQNPLALVSRHHLHHLPRTSSSSRGQFSQKRDFATSSSKPS
NCU03877.1	------------------------------------------------------------
FG10267.1 	------------------------------------------------------------
AN2998.1  	------------------------------------------------------------
          	                                                            

MG01014.1 	SDMAAIKIDGTAIAKKIRERLHAEIDEKQAVNPHYKPCLNIIQV1GDRSDSS1TYVRMKL
NCU03877.1	---MASKIDGTALAKRIRDGLRATIDETKKTNPRFRPCLKIIQV1GDRSDSS1TYVRMKL
FG10267.1 	--MVATKLDGNAIAKSIREKLCAEVNEKQKINPRFKPCLKIIQA1-------~TYVRMKL
AN2998.1  	--------------------------------------------~-------~-------
          	                                                            

MG01014.1 	KAAEE0AGIQCELLHYPETISEAELLNQLHRINNDPNIHGVLVQLPLPKHLDEYAVTSAV
NCU03877.1	KAAEE0CGIEAELIKFDENATEAELVAKVVQLNNDSTVHGMLVQLPLPKHISEYAVTSAV
FG10267.1 	KAAAE0AGIDSQLLHYPESISEAELLDHIRQHNNDPAVHGILVQLPLPKHISEYTVTSFV
AN2998.1  	-----~------------------------------------------------------
          	                                                            

MG01014.1 	ADEKDVDGFGATNIGELAKKGGRPYFIPCTPKGIMVLLEEAGVDLKGKNAVVLGRSDIVG
NCU03877.1	ADEKDVDGFGTLNIGELAKRGGHPYFIPCTPKGVMALLAEAGVELKGKNAVVLGRSDIVG
FG10267.1 	ADEKDVDGFGTKNIGELAKRGGKPLFIPCTPKGCMILLKESGIDLKGKNAVVLGRSDIVG
AN2998.1  	------------------------------------------------------------
          	                                                            

MG01014.1 	SPVSYLLKNADATVTVCHSRTVGLQDHLKAADIVVAAIGKAGFVKGEWLKPGAVVIDVGT
NCU03877.1	SPVSYLLKNADATVTVCHSKTADLQQYLKEADVVVAAIGQPNFVKGEWLKKGAVVIDVGT
FG10267.1 	SPVSYLLRNADATVTVVHSKTQNIEQHVRNADVLVAAIGQPLFVPGSWIKPGAVVIDVGT
AN2998.1  	------------------------------------------------------------
          	                                                            

MG01014.1 	NYIPDASKKSGQRLVGDVDFESAAQVASHITPVPGGVGPMTVAMLLQNVVDSATMHTQRQ
NCU03877.1	NYVPDDTKKSGVRLVGDVEFESASQVASAITPVPGGVGPMTVAMLLQNVVDATSFYFENQ
FG10267.1 	NFLPDATKKSGHRLVGDVDYASAVEVASAITPVPGGVGPMTVAMLMQNVVDATTWYFESQ
AN2998.1  	---------------------------------------MTVAMLLENVVASAKAYFEKQ
          	                                       ******::*** ::. : : *

MG01014.1 	KNRKTVPLPLKLLDPVPSDIAISRAQVPKQIVRVAEEVGIGASELEPYGAYKAKVDLSLL
NCU03877.1	KKRGSIPLPLKLAEPVPSDIDISRAQVPKPITQIATEIGLAPSELEPYGAYKAKVDLSIL
FG10267.1 	KLRKTIPLPLKLEDPVPSDIAVSRAQTPKDITRIAAEIGIAPHELEPYGAYKAKVDLGLM
AN2998.1  	KERHITPLPLKLATPVPSDIAISRSQYPKPITQVASEIGIASHELEPYGHTKAKVSLEVL
          	* *   ******  ****** :**:* ** *.::* *:*:.. ******  ****.* ::

MG01014.1 	DRLKHRRNGRYVVVTGITPTPLGEGKSTTTMGIAQALGGHLGRPTFANVRQPSQGPTFGI
NCU03877.1	KRLEHRRNGRYVVVTGITPTPLGEGKSTTTMGLAQALGAHVGRLTFANVRQPSQGPTFGI
FG10267.1 	QRLEHRRNGRYVVVTGITPTPLGEGKSTTTMGLAQALGAHVGRMTFANVRQPSQGPTFGI
AN2998.1  	NRLSHRRNGRYILVCGITPTPLGEGKSTTTLGLSQALGAHLNRVAFANVRQPSQGPTFGI
          	.**.*******::* ***************:*::****.*:.* :***************

MG01014.1 	KGGAAGGGYSQVIPMDEFNLHLTGDIHAITAANNLLAAAIDTRIFHENTSKDAGLFKRLV
NCU03877.1	KGGAAGGGYSQVIPMDEFNLHLTGDIHAITAANNLLAAAIDTRMFHENTQKDGPLYKRLV
FG10267.1 	KGGAAGGGYSQVIPMDEFNMHLTGDIHAIGAANNLLAAAIETRIFHENTQKDGPLYKRLV
AN2998.1  	KGGAAGGGYSQVIPMDEFNLHLTGDIHAITAANNLLAAAIETRMFHEATQKDAALYKRLV
          	*******************:********* **********:**:*** *.**. *:****

MG01014.1 	PVKNGKREFAPIMFRRLKKLGITKTDPAELTEEEISRFVRLDIDPETITWRRVLDVNDRH
NCU03877.1	PAKNGKREFPPIMFRRLKKLGIEKTNPDDLTEEEISRFARLDIDPETITWRRVLDVNDRH
FG10267.1 	PVKNGARKFAPVMFRRLKKLGIDKTNPDELTEDEIHRFARLDIDPETITWRRVLDVNDRH
AN2998.1  	PEKKGKREFKPIMFKRLKKLGINKTDPNELTEEEINRFARLDIDPSTITWRRVLDVNDRH
          	* *:* *:* *:**:******* **:* :***:** **.******.**************

MG01014.1 	LRGITIGTAPTEKGQTRESGFDISVASECMAILALSTSLADMRERLGSMVVATSRSGDPV
NCU03877.1	LRGIVIGNAPTEKGHKRDTGFDISVASECMAILALSTSLADMRERLGRMVVASSKSGDPV
FG10267.1 	LRGITVGAAPTEKGQTRETGFDITVASECMAILALSNNLAEMRERLGSMVVATSRGGDPV
AN2998.1  	LRGITVGQAPTEKGLTRETGFDISVASECMAILALSSDLADMRERLGRMVVATSKRGEPV
          	****.:* ****** .*::****:************..**:****** ****:*: *:**

MG01014.1 	TCDDIGAGGALTALMRDAIKPNLMQTLEGTPVFVHAGPFANISIGNSSILADKIALKLVG
NCU03877.1	TADDLGVGGALTVLMRDAIKPNLMQTLEGTPVFVHAGPFANISIGNSSILADKMALKLAG
FG10267.1 	TADDLGAGGALTALMKDAIKPNLMQTLEGTPVFVHAGPFANISIGQSSILADKLALKLAG
AN2998.1  	TCDDIGAGGALAALMKDAIKPNLMQSLEGTPVLVHAGPFANISIGASSVLADRVALKLAG
          	*.**:*.****:.**:*********:******:************ **:***::****.*

MG01014.1 	TEPDEDHSANTGFVVTEAGFDFTMGGERFFNIKCRTSGLVPDVVVIVATVRAIKVHGGGP
NCU03877.1	TEPDEDYATKSGFVVTEAGFDFTMGGERFFNIKCRTSGLMPDVVVVVATIRALKVHGGGP
FG10267.1 	TEADEDHQKKAGFVVTEAGFDFTMGGERFFNIKCRTSGLVPDVVVIVATVRALKVHGGAP
AN2998.1  	TEPEEDHEAKTGFVVTEAGFDFTMGGERFFNIKCRSSGLSPDTVVIVATVRALKVHGGGP
          	**.:**:  ::************************:*** **.**:***:**:*****.*

MG01014.1 	PITPGAPLSPVYKEENVETLRAGCTNLRKQIENAKQYGVPVVVAINKFSTDTDAEVAVVR
NCU03877.1	PISPGAPLNPVYKEENVEILRAGCVNLAKHIQNARSYGVPVVVAINKFSTDTQAEIDVVR
FG10267.1 	PIAPGAALSPVYKEENVDILRAGCVNLKKQIANAKSFGIPVVVAINKFATDTDAEIAVIR
AN2998.1  	EISPGAPLHEVYRTENTEILRKGCVNLKKHIENARQYGVPVVVAINRFETDTEAEIAIIR
          	 *:***.*  **: **.: ** **.** *:* **:.:*:*******:* ***:**: ::*

MG01014.1 	EESLAAGAVDAVLSNHWAEGGKGAIDLAQAVIAASDLPKPD-FKLLYSLEG-TVQERIEK
NCU03877.1	EEAIRAGAEDAVLANHWAEGGKGAVDLAQAVISASEKSTPETQKLLYEVEGNTVQERIEK
FG10267.1 	EEAVSAGAENAILSNHWAEGGKGAVDLAHGVIAAAE--KPKDLKFTYNLEG-TVQERLEA
AN2998.1  	EEAISAGAEDAVSANHWAEGGAGAVDLAKAVIIAS--SKPKDFKLLYDLNG-SIQERIER
          	**:: *** :*: :******* **:***:.** *:  ..*.  *: *.::* ::***:* 

MG01014.1 	IAKDMYGAAAVEFSELAQKKVETYTRQGFGNLPICIAKTQYSTSHDPELKGAPKGFTVPI
NCU03877.1	IACEMYGAASVEFSELAQKKVDTYTRQGFGNLPICIAKTQYSLSHDPELKGAPTGFTVPI
FG10267.1 	IGKQMYGAEKVELSELAQKKVDTYTRQGFGHLPICVAKTQYSLSHDPDLKGAPTGFTVPI
AN2998.1  	IGKAMYGAEKVEFSELAQKKVDTYTAQGFSNLPICIAKTQYSLSHDPALKGAPTGFTVPI
          	*.  ****  **:********:*** ***.:****:****** **** *****.******

MG01014.1 	RDVRMAAGAGYL2YALAADIQTIPGLPTAPGYLNVDVDLETGEIDG~LF-----------
NCU03877.1	RDVRMAAGAGYL~YALAADIQTIPGLPTAPGYFNVDVDTETGEIQG~LF-----------
FG10267.1 	RDVRLAAGAGYF2YALAADIQTIPGLPTAPGYLNVDVNVETGEIEE1CSTAATKSRAYDA
AN2998.1  	RDVRLAVGGGYL2YALAADIQTIPGLPTAPGYLNVDIDPETGEIDG~LF-----------
          	****:*.*.**: *******************:***:: *****:               

MG01014.1 	---~-----------------------------~-------------~------------
NCU03877.1	---~-----------------------------~-------------~------------
FG10267.1 	GNM2PVIQLKVYRMGTTDSKLTIGRNRIATRSH0GERRPLLDTNTYP1NKLGSTSISPDV
AN2998.1  	---~-----------------------------~-------------~------------
          	                                                            

MG01014.1 	~--------------------
NCU03877.1	~--------------------
FG10267.1 	1TTEEFNPTNGSDLSLSVRYG
AN2998.1  	~--------------------
          	
```
